# Gene expression data analysis identifies multiple deregulated pathways in patients with asthma

**DOI:** 10.1042/BSR20180548

**Published:** 2018-11-07

**Authors:** Reem H. Alrashoudi, Isabel J. Crane, Heather M. Wilson, Monther Al-Alwan, Nehad M. Alajez

**Affiliations:** 1 Clinical Laboratory Science, College of Applied Medical Science, King Saud University, Riyadh 11461, Kingdom of Saudi Arabia; 2 Infection, Immunity and Inflammation, School of Medicine, Medical Sciences and Nutrition, University of Aberdeen Institute of Medical Sciences, Aberdeen, U.K.; 3 Stem Cell and Tissue Re-Engineering Program, King Faisal Specialist Hospital and Research Centre, Riyadh, Saudi Arabia; 4 College of Medicine, Al-Faisal University, Riyadh, Saudi Arabia; 5 Stem Cell Unit, Department of Anatomy, College of Medicine, King Saud University, Riyadh 11461, Kingdom of Saudi Arabia; 6 Cancer Research Center, Qatar Biomedical Research Institute, Hamad Bin Khalifa University (HBKU), Qatar Foundation, Doha, Qatar

**Keywords:** asthma, allergy, gene expression

## Abstract

Asthma is a chronic inflammatory disorder associated with airway hyper-responsiveness. Although a number of studies have investigated asthma at the molecular level, the molecular immune signatures associated with asthma severity or with the response to corticosteroids are still being unraveled. The present study integrated four asthma-related gene expression datasets from the Gene Expression Omnibus and identified immune-gene signatures associated with asthma development, severity, or response to treatment. Normal and mild asthmatic patients clustered separately from the severe asthma group, suggesting substantial progression-related changes in gene expression. Pathway analysis of up-regulated severe asthma-related genes identified multiple cellular processes, such as polymorphism, T-cell development, and transforming growth factor-β signaling. Comparing gene expression profiles of bronchoalveolar lavage cells in response to corticosteroid treatment, showed substantial reductions in genes related to the inflammatory response, including tumor necrosis factor signaling in the corticosteroid sensitive versus resistant patients, suggesting a defective immune response to corticosteroids. The data highlight the multifactorial nature of asthma, but revealed no significant overlap with the gene expression profiles from different datasets interrogated in current studies. The presented profile suggests that genes involved in asthma progression are different from those involved in the response to corticosteroids and this could affect the clinical management of different groups of patients with asthma.

## Introduction

Asthma is a chronic inflammatory disorder characterized by reversible airway obstruction and hyperresponsiveness [1]. Asthma can be a long-lasting inflammation since childhood or a disease that develops later in life. Many patients will have a genetic predisposition to developing asthma. Allergen exposure can cause an inflammatory condition mediated by a complex interaction between innate and adaptive immune cells. The initial description of asthma involves identifying clinical, physiological, and cellular parameters. Clinical characteristics include age at asthma onset, allergen type, family history, or even the degree of airway obstruction; the familiar physiological features, such as wheezing, shortness of breath, and coughing; and cellular parameters [2]. These characteristics alone are not enough to identify the importance of a specific pathological process or improve the treatment of patients with asthma.

Asthma remains a major health problem because it is currently one of the most common chronic disorders worldwide [3]. The prevalence of asthma varies in different geographical locations, with the highest prevalence in countries such as Australia (21.5%), Sweden (20.2%), U.K. (18.2%), Netherlands (15.3%), and Brazil (13.0%). The global prevalence of diagnosed asthma in adults has been estimated as 4.3%, ranging from 0.2% in China to 21.0% in Australia [4,5]. Although asthma prevalence is higher in developed countries, the incidence of asthma in developing countries is increasing [6].

Previous literature reviews revealed more than 120 and 150 genes that were related to asthma in human and animal models, respectively [7,8]. These findings indicated that asthma is a polygenic disease and its intricacy originates from interactions between several genes and environmental factors. This interaction leads to consecutive changes in the airway microenvironment, starting with the stimulation of inflammatory pathways and recruitment of immune cells that are not usually present in the airway, leading to bronchoconstriction, airway hyper-responsiveness, and airway remodeling [7]. The advantage of gene analysis is that it allows the detection of asthma at an early stage and helps to predict disease progression [9]. Therefore, primarily analyzing several publicly available studies related to gene expression changes in mononuclear cells could allow a more global view of the changes in the expression of genes related to mononuclear cells in asthma. Monocytes play an important role in immune and inflammatory diseases, and while they have not been identified as key players in asthma, they are likely to be pro-inflammatory, be readily recruited to tissues, and have a major role in the exacerbation of the disease. Allergens, one of the main factors that can trigger and exacerbate asthma, include a wide range of indoor allergens, such as dust mites, cockroaches, fungi, and furred animals; and outdoor allergens, such as pollens and molds. In the present study, we analyzed several publicly available gene expression datasets related to asthma and identified differential gene signatures that are associated with asthma progression and those that are related to the response to corticosteroids.

## Materials and methods

### Microarray data analysis

Using monocyte and asthma search terms and limited to *Homo sapiens* studies, the GSE27876, GSE7368, GSE19301, and GSE40240 raw gene expression datasets were retrieved from the Gene Expression Omnibus GEO, based on the inclusion/exclusion criteria (peripheral blood mononuclear cells, expression profiling experiment type, and patients with asthma) and were imported into GeneSpring 13.0 software (Agilent Technologies, Palo Alto, CA, U.S.A.). Raw data were subsequently normalized using the percentile shift, and a 2.0 (for GSE27876 and GSE7368) and 1.5 (for GSE19301 and GSE40240) fold-change (FC) cutoff, and *P*<0.05 were used to determine significantly changed transcripts between groups. Details of the fours datasets included in the present study are listed in Table 1. Clinical data associated with patients from those studies have been described previously [10,12,13].

**Table 1:** Details of the four datasets included in the present study

Series	Title/Link	Experiment type	Experiment design	Investigation
GSE27876	*In vivo* and *in vitro* study of asthma http://www.ncbi.nlm.nih.gov/geo/query/acc.cgi?acc=GSE27876 (Lee et al., 2012)	Expression profiling by array	To induce an inflammatory response, for *in vitro* study, THP1 cells were stimulated for 24 h with LPS or HDM extract. After stimulation, THP1 cells were harvested for total RNA extraction. Total RNA was extracted from the buffy coat fractions and RNA was reverse transcribed into cDNA for microarray analysis.	Analysis of gene expression patterns of peripheral blood cells from asthma patients compared with those from normal subjects using microarray analyses.
GSE19301	Gene expression patterns in peripheral blood mononuclear cells associated with asthma exacerbation attack http://www.ncbi.nlm.nih.gov/geo/query/acc.cgi?acc=GSE19301 (Bjornsdottir et al., 2011)	Expression profiling by array	PBMCs collected during (a) quiet (*N* = 394 samples from 118 subjects), (b) exacerbation (*N* = 166 samples from 118 subjects), and (c) follow-up (*N* = 125 samples from 102 subjects).	Gene expression changes in PBMCs associated with asthma exacerbations over the course of a year. Gene expression levels during stable asthma, exacerbation, and 2 weeks after an exacerbation were compared.
GSE7368	Corticosteroid resistant asthma is associated with classical activation of airway macrophages and exposure to LPS http://www.ncbi.nlm.nih.gov/geo/query/acc.cgi?acc=GSE7368	Expression profiling by array	Asthmatics had a baseline FEV1% predicted of 55–85%, asthmatic patients were defined as CS if they had an increase in FEV1% predicted of greater than 15% after a 1-week course of prednisone, and as CR if less than 12% change in FEV1% predicted was observed.	Gene microarray analysis was performed with bronchoalveolar lavage (BAL). The cause of corticosteroid resistant (CR) asthma is unknown. Gene microarray technologies have the potential to substantiate new hypotheses regarding the etiology of corticosteroid resistance.
GSE40240	Allergen inhalation challenge effect on peripheral blood of asthmatic early responders (ERs) and dual responders (DRs) (Singh et al., 2013)	Expression profiling by array	Blood was collected immediately prior to, and 2 hours after challenge. The change in gene expression (post-expression minus pre-expression) in ERs was compared with the change in gene expression in DRs using age and sex as covariates.	Analysis of blood collected from asthmatics immediately prior to, and 2 hours after, cat allergen challenge. Some asthmatics are early responders while others are dual (early plus late) responders.

### Statistical analysis

Pathway analyses were conducted using the Database for Annotation Visualization and Integrated Discovery (DAVID) functional annotation and clustering bioinformatics tool, as described in our previous report [14]. Statistical analyses and graphing were performed using GraphPad Prism 6.0 software (GraphPad Software, San Diego, CA, U.S.A.).

## Results

### Identification of the gene signature associated with asthma progression

**Figure 1:**
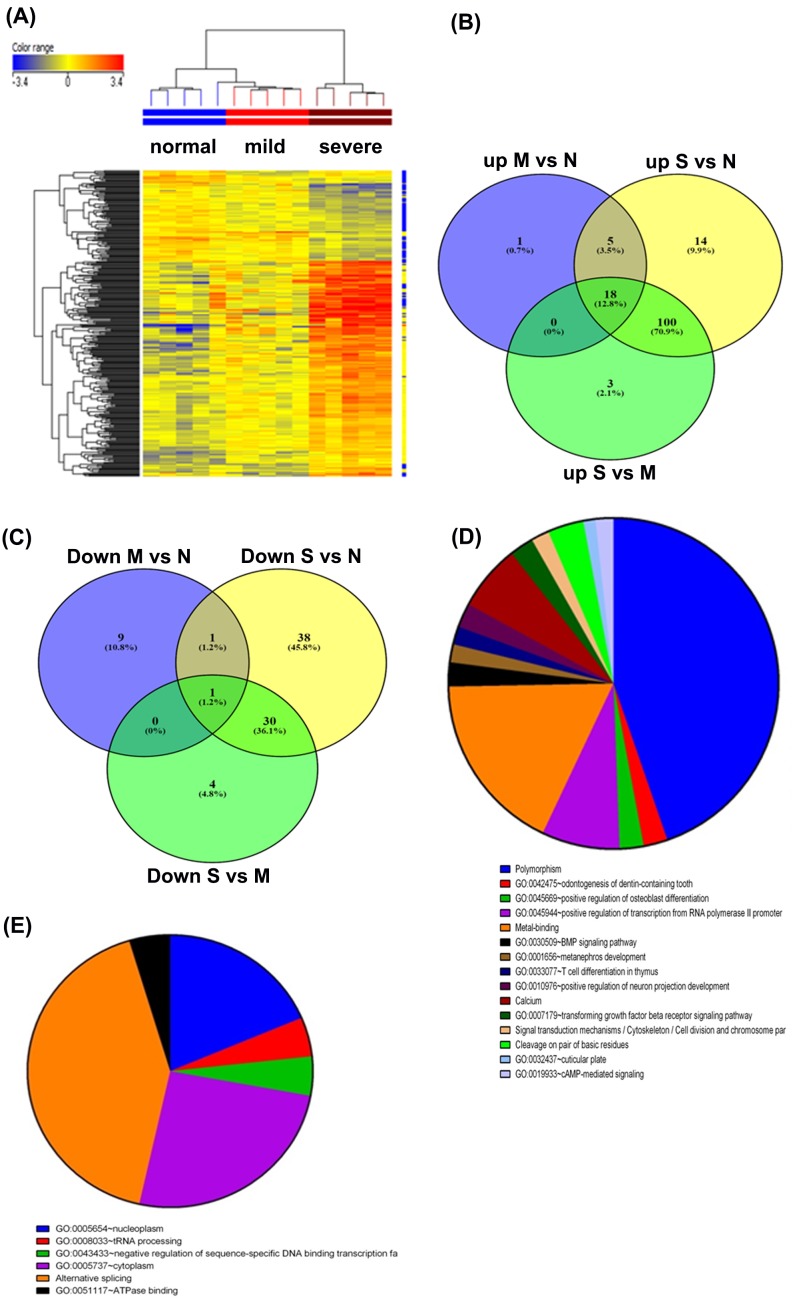
Identification of the gene signature associated with asthma development GSE27876 series: (**A**) Hierarchical clustering based on differentially expressed mRNAs for patients with mild, moderate, and severe asthma. (**B** and **C**) Comparisons of the up-regulated and down-regulated genes between Mild (M) or Severe (S) versus Normal (N), and Mild versus Severe. (**D**) Functional interpretation of the 118 up-regulated genes related to asthma severity. (**E**) Functional interpretation of the 31 down-regulated genes related to asthma severity, and enrichment in six categories.

First, we analyzed the GSE27876 series comparing peripheral blood cells from mild and severe asthma that were selected from patients classified into the asthma treatment step 4, based on the criteria described in the Global Initiative for Asthma (GINA) (in the present study the selected severe asthma patients had medication records of high-dose steroid inhalation with no oral medicine) compared with those from normal subjects [10]. Using a 2-fold change cut-off, gene expression data analysis revealed 311 differentially expressed mRNA transcripts across all samples. Hierarchical clustering based on the differentially expressed mRNAs revealed clear separation of the severe from the mild and the mild asthmatic from the normal control group (Figure 1A). Interestingly, we observed that the normal and mild asthma groups clustered together while the severe asthma group clustered away from the other two groups (Figure 1A). Further analysis revealed 24 up-regulated and 11 down-regulated genes in patients with mild asthma compared with normal individuals (Figure 1B,C). Further differentially expressed genes were detected in patients with severe asthma, with 137 up-regulated and 69 down-regulated genes compared with normal individuals (Figure 1B,C). Further analysis revealed the up-regulation of 121 genes and down-regulation of 35 genes in patients with severe compared with mild asthma (Figure 1B,C). When comparing the differentially expressed genes in severe versus control and severe versus mild asthma groups, 100 genes were found to be up-regulated, while 30 genes were down-regulated (Figure 1B,C; Tables 2 and 3). Interestingly, 18 genes were found to be up-regulated in the severe versus normal, mild versus normal, and severe versus mild comparisons (Figure 1B,C and Table 2). There were common genes between these two study groups, where five genes were up-regulated (*ATP5E, BRK1, KCNJ6, RNASEH2C*, and *RPL9*) and one gene (*RAB5B*) was down-regulated in patients with mild and severe asthma compared with normal individuals (Tables 2 and 3). Functional annotation using the DAVID gene functional classification tool conducted on the 118 up-regulated genes related to asthma severity revealed multiple enriched functional categories, including polymorphism, T-cell development, and transforming growth factor-β (TGF-β) signaling (Figure 1D). The most highly enriched functional category was polymorphism. Functional annotation conducted on the 30 down-regulated genes related to asthma severity identified significant enrichment in six categories (Figure 1E).

**Table 2:** Common up-regulated genes between different asthmatic groups from the GSE27876 dataset

**(a) List of the common up-regulated genes between the severe versus normal and severe versus mild groups**				
*ACSM2A*	*ADCYAP1*	*ANKRD20A2*	*ARCN1*	*BOLL*
*CAMK1G*	*COL25A1*	*COL3A1*	*CPA2*	*CPLX1*
*CYP19A1*	*DCST2*	*DIO1*	*DMD*	*DPY19L2P2*
*DYDC2*	*DYRK3*	*ECT2*	*EEF1E1*	*EFCAB11*
*EFHB*	*ERVW-1*	*ESR1*	*ESRRG*	*EYA1*
*FAM131C*	*FAM170A*	*FGF20*	*FNDC7*	*FZD5*
*GDF9*	*GDNF*	*GJA3*	*GJA5*	*GLI3*
*GNRH2*	*GPR115*	*GRIK2*	*GULP1*	*GUSBP3*
*HOXC12*	*IKZF4*	*KNCN*	*KRTAP4-1*	*LCA5L*
*LINC00466*	*LINC01133*	*lnc-ARSJ-1*	*lnc-FAM*	*lnc-PPIAL4G-9*
*lnc-PTN-2*	*LOC101060510*	*LOC101929315*	*MAP7D2*	*MAP9*
*MARK2*	*MOB3B*	*MTIF2*	*NAPG*	*NPAS2*
*NPR3*	*NUCKS1*	*OGG1*	*PCDH7*	*PINK1-AS*
*PPIC*	*PPP1R16A*	*RAB39B*	*RAG1*	*RPL23A*
*RPS27*	*RRP15*	*RUNX2*	*RWDD4*	*SEZ6*
*SIAE*	*SKIL*	*SLC19A3*	*SLC26A7*	*SNAPC4*
*SPAG16*	*SPTA1*	*SPX*	*TARID*	*TDRD5*
*TENM3*	*TGM2*	*TMEM213*	*TPT1*	*TRIM42*
*UBE2S*	*VSNL1*	*VTA1*	*XKRY2*	*XLOC_l2*
*YAP1*	*ZDHHC13*	*ZNF385B*	*ZNF396*	*ZNF562*
**(b) List of the common up-regulated genes between the severe versus normal, mild versus normal, and severe versus mild groups**				
*ACVR2B*	*AKR1B10*	*ANXA8L1*	*BMPR1A*	*CADPS*
*CFH*	*FAM110D*	*FAM74A6*	*HS3ST5*	*JAM2*
*KLK7*	*LOC101927000*	*MYOM3*	*P2RX6P*	*RBMS3*
*SORBS1*	*SPIN1*	*TFF1*		
**(c) List of the common up-regulated genes between the severe versus normal and mild versus normal groups**				
*ATP5E*	*BRK1*	*KCNJ6*	*RNASEH2C*	*RPL9*

**Table 3:** Common down-regulated genes between different groups of patients with asthma from the GSE27876 dataset

**(a) List of the common down-regulated genes between the severe versus normal and severe versus mild groups**				
*ALG3*	*BLOC1S5*	*C10orf95*	*CRLF3*	*DEXI*
*ELAC2*	*FBL*	*IPO5P1*	*KDM5B*	*lnc-AC040934.1-3*
*LOC155060*	*LOC440311*	*LRRC37A4P*	*MRPS34*	*MYO15B*
*NME6*	*PDPR*	*PURA*	*RAB4B*	*RARRES3*
*RNF157*	*RPS6KA5*	*RPTOR*	*SERGEF*	*SIGIRR*
*SNTA1*	*SRSF6*	*SUFU*	*TMEM186*	*ZMYM6*
**(b) List of the common down-regulated genes between the severe versus normal, mild versus normal, and severe versus mild groups**				
*SPOCK2*				
**(c) List if the common down-regulated genes between the severe versus normal and mild versus normal groups**				
*RAB5B*				

### Identification of gene signatures associated with corticosteroid sensitivity

**Figure 2:**
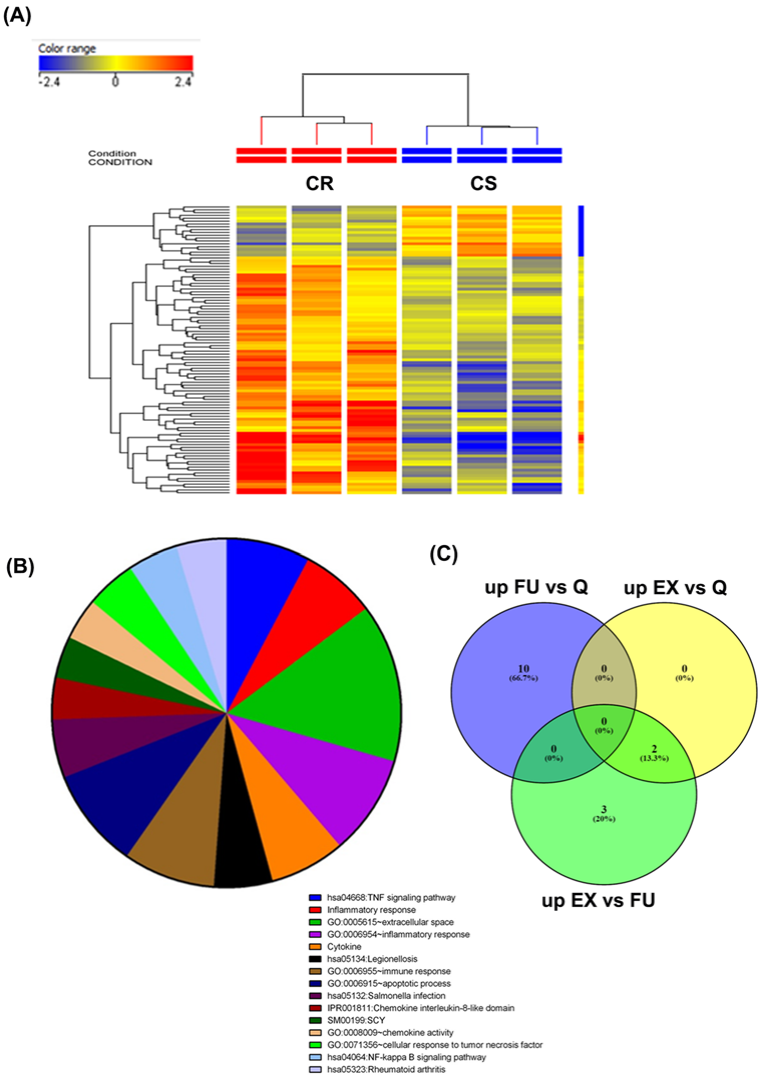
Identification of the gene signature associated with asthma exacerbation GSE7368 series: (**A**) Hierarchical clustering based on differentially expressed mRNAs in response to corticosteroids. (**B**) Functional interpretation of the 70 up-regulated genes related to corticosteroid resistance. (**C**) The common up-regulated genes between GSE19301 series groups; EX, exacerbation; FU, follow-up; Q, quiet.

We subsequently analyzed the GSE7368 series comparing bronchoalveolar lavage (BAL) cells from corticosteroid resistant (CR) and corticosteroid sensitive (CS) patients with asthma. The cells consisted mainly of macrophages, neutrophils, and lymphocytes. Patients were defined as CS if they had an increase in forced expiratory volume 1 (FEV1) predicted (which is defined as the FEV1 of the patient divided by the average FEV1 in the population for any person of similar age, sex, and body composition) of greater than 15% after a 1-week course of prednisone, and as CR if a less than 12% change in FEV1 predicted was observed [11]. Hierarchical clustering based on differentially expressed mRNAs (2.0 FC) revealed clear separation of the CR from the CS groups (Figure 2A). Further analysis identified 70 up-regulated and 16 down-regulated genes in the CR patients compared with the CS patients (Table 4). The up-regulated genes were further classified based on functional clustering using DAVID. BAL cells from CS patients showed significant enrichment in several pathways related to inflammatory responses, such as tumor necrosis factor (TNF) signaling pathways, inflammatory responses, cytokines, immune responses, chemokines, interleukin-8-like domain, and the nuclear factor kappa B (NF-κB) signaling pathway (Figure 2B).

**Table 4:** Differentially expressed genes related to corticosteroid resistance from the GSE7368 study

**(a) List of the up-regulated genes in the corticosteroid resistance versus the corticosteroid sensitive groups**				
*CCL3L3*	*AC079305.10*	*ADM*	*AKAP2*	*BPIFB1*
*CAHM*	*CCL20*	*CCL3*	*CCL4*	*CD2*
*CHI3L1*	*CTA-29F11.1*	*CXCL1*	*CXCL2*	*CXCL3*
*DUSP2*	*EGR1*	*EIF4E*	*EPC1*	*FAM46C*
*FOSB*	*GBP2*	*GPX3*	*HSPA1A*	*ICAM1*
*IER2*	*IER3*	*IL1α*	*IL1β*	*IL6*
*IL8*	*KLF6*	*KLRB1*	*LBH*	*LINC00936*
*LOC100129518*	*LOC100506098*	*LOC101060267*	*LPXN*	*MT1G*
*MT1H*	*MT1X*	*NFKBIA*	*NFKBIE*	*NFKBIZ*
*NFKBIZ*	*NR4A1*	*OTUD1*	*PADI2*	*PERP*
*PHLDA1*	*PIGR*	*PLEKHA5*	*PMAIP1*	*PPP1R15A*
*PPP1R15A*	*PSD3*	*RABGAP1L*	*SCGB1A1*	*SERPINB3*
*SERPINB3*	*SLPI*	*SNORD3A*	*TACSTD2*	*THAP2*
*TMC5*	*TMEM107*	*TNF*	*TNFAIP3*	*TRA2B*
*ZFP36L1*				
**(b) List of the down-regulated genes in the corticosteroid resistance versus the corticosteroid sensitive groups**				
*ABHD2*	*BCAT1*	*CBS*	*CCL24*	*CDKN1B*
*DENND4C*	*HHEX*	*IRAK3*	*IREB2*	*NXPE3*
*PAG1*	*PKN2*	*RPS24*	*SH3PXD2B*	*SOCS1*
*TMEM170B*				

### Identification of gene signature associated with asthma exacerbation

To identify the gene signature associated with asthma exacerbation, the GSE19301 series comparing the gene expression profile in peripheral blood mononuclear cells (PBMCs) associated with an asthma exacerbation attack was analyzed. For the GSE19301 series, samples were collected while the subjects were experiencing one or more of the following symptoms: increases in wheezing, chest tightness, and/or shortness of breath. Gene expression levels during stable (quiet) asthma, exacerbation, and 2 weeks after an exacerbation were compared [12]. The common up-regulated genes between ‘Exacerbation versus Quiet’ and ‘Exacerbation versus Follow-up’ were the genes that encode interferon α-inducible protein 27 (*IFI27*) and interferon-induced protein 44 Like (*IFI44L*) [15] (Figure 2C). Down-regulated genes were only observed in the ‘Exacerbation versus Follow-up’ group, and they were lactotransferrin (*LTF*) and the defensin α 1 family (*DEFA1, DEFA1B*, and *DEFA*).

### Identification of the gene signature associated with early versus dual responders to inhalers

**Figure 3:**
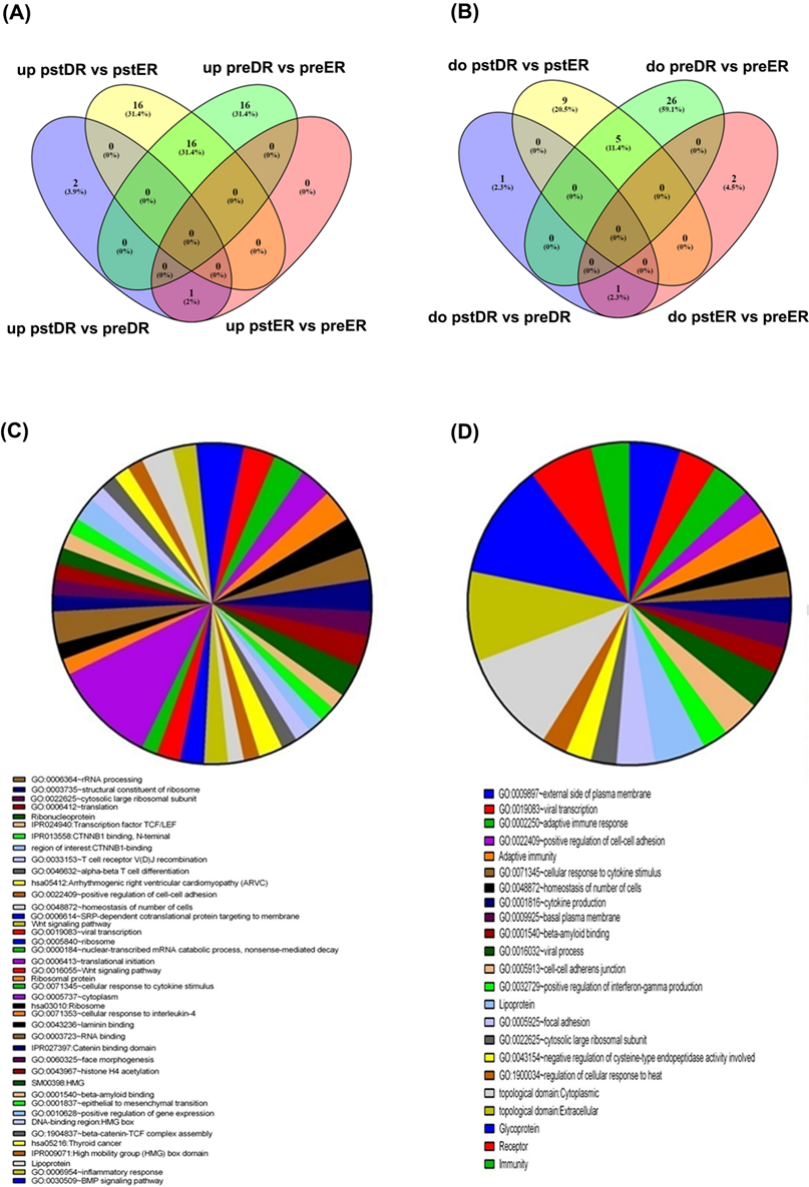
Identification of the gene signature associated with early versus dual responders to inhalers GSE40240 series. (**A** and **B**) The common up-regulated and down-regulated genes between the GSE40240 series groups. (**C**) Functional interpretation of the 32 up-regulated genes related to immediate response following allergen challenge. (**D**) Functional interpretation of the 32 up-regulated genes related to a delayed response following allergen challenge; pstDR, post dual responders; pstER, post early responders; preDR, pre dual responders; preER, pre early responders.

To identify the gene signature that could discriminate early from late responders to inhalers, the GSE40240 series comparing the peripheral blood cell gene expression profile from asthmatic early responders (ERs) to dual (early plus late) responders (DRs) following allergen inhalation challenge was analyzed. Study participants were classified as ERs if the initial drop in FEV1 resolved back to baseline within 1–3 h of allergen inhalation and if the maximum drop in FEV1 between 3 and 7 hours was less than 15%. Study participants were classified as DRs if, in addition to the early response, the participants experienced a maximum drop in FEV1 of 15% or greater between 3 and 7 h of allergen inhalation. In this series, blood samples were collected from 28 subjects immediately before and 2 h after challenge, and the changes in gene expression (post-exposure versus pre-exposure) in ERs were compared with the changes in gene expression in DRs, using age and sex as covariates [13]. Analysis of basal gene expression before inhalation challenge identified 32 genes that were up-regulated in the DR compared with the ER group. Interestingly, 2 h after challenge, only 16 out of the 32 genes were still elevated in the DR versus ER group. Sixteen additional genes were also up-regulated in the post-exposure DR versus post-exposure ER groups (Figure 3A and Table 5). By contrast, 31 genes were down-regulated immediately before allergen challenge in the DR group compared with the ER group. However, 2 h later, only five genes were still down-regulated and an additional nine genes became down-regulated in the DR compared with the ER groups (Figure 3B and Table 5). In the DR group post-allergen challenge, three genes were up-regulated and two genes were down-regulated compared with pre-allergen challenge (Figure 3A,B), while in the ER group post-allergen challenge, one gene was up-regulated and three genes were down-regulated compared with before allergen challenge. In the present study, the direct response to allergen challenge led to up-regulation of 32 genes in the DR group compared with the ER group. Conducting functional clustering on these genes using DAVID showed enrichment in many categories. Most of the pathways were not related to inflammatory responses except for three categories: cellular response to IL-4, cellular response to cytokine stimulus, and cellular response to inflammation (Figure 3C). Similar findings were observed in the delayed response. The functional classification of the 32 up-regulated genes in the DR group compared with the ER group after 2 h of allergen response showed that these down-regulated genes were associated with different signaling pathways; some of them were related to inflammation and the adaptive immune response (Figure 3D).

**Table 5:** Differentially expressed genes between early (ER) and dual responders (DR) from the GSE40240 dataset

**(a) List of the common up-regulated genes between ‘up post DR versus post ER’ and ‘up pre DR versus pre ER’ group**				
*C12orf57*	*CAMK4*	*CCR7*	*GCNT4*	*ITGA6*
*ITM2A*	*LEF1*	*LOC100134091*	*MAL*	*RPL30*
*RPL41*	*RPL4P4*	*THEMIS*	*TRABD2A*	*VSIG1*
*ZNF83*				
**(b) List of the common up-regulated genes between ‘up post DR versus pre DR’ and ‘up post ER versus pre ER’**				
*MME*				
**(c) List of the common down-regulated genes between ‘down post DR versus post ER’ and ‘down pre DR versus pre ER’ groups**				
*LILRA3*	*CYP1B1*	*PI3*	*PISD|MIR7109*	*TREML4*
**(d) List of the common down-regulated genes between ‘down post DR versus pre DR’ and ‘down post ER versus pre ER’**				
*DDIT4*				

Abbreviations: DR, dual responders; ER, early responders.

## Discussion

In the present study, we interrogated several publicly available datasets and identified differentially expressed genes in immune cells correlating with asthma, severity, response to allergens, and response to corticosteroid treatment. Our analysis revealed a large number of genes that were either up-regulated or down-regulated in patients with asthma in comparison with the control group or other groups of patients that were also investigated within the same study, depending on the study design. While each dataset revealed distinct gene expression patterns, we observed minimal overlap among the different studies. This was not surprising because each study dealt with a different aspect of asthma (such as asthma progression versus response to corticosteroid treatment). Additionally, it is plausible that several of the observed differences may also due to the different experimental design of the databases studied: The kind of material analyzed (BAL and PMCs) and size of the samples studied. Therefore, our data suggested that asthma is a multifactorial disease where distinct factors can be engaged in the development of asthma, its severity, or the response to treatment.

In the GSE27876 dataset, changes in gene expression in patients with mild and severe asthma compared with normal individuals were assessed and revealed clear associations between changes in gene expression and asthma severity. More genes correlating with asthma severity were up-regulated than down-regulated. Interestingly, patients with mild asthma did not have as many changes in gene expression when compared with normal controls. While few of these deregulated genes were common between patients with mild and severe asthma, more affected genes were observed with asthma severity. However, when the patients with severe asthma were compared with normal individuals or patients with mild asthma, the differentially expressed genes were mostly the same for both groups, which implied that changes in gene expression are mostly affected by severity. Functional annotation of the differentially expressed genes in patients with severe asthma highlighted a plausible role for genes related to polymorphism, T-cell development, and TGF-β signaling in the severity of asthma. Relatively recent study has reported a single nucleotide polymorphism rs848 in the IL13 gene region that was significantly associated with asthma severity in patients that were lifetime physician-diagnosed as asthmatic in Italy [16].

The aim of the GSE7368 study was to compare the changes in gene expression between the CR and the CS asthma patients in response to corticosteroid treatment. The level of change in gene expression in the present study correlated with CR; in addition, more genes were up-regulated than down-regulated in the CR patients compared with the CS patients. This finding implied that the CR patients did not respond to corticosteroid treatment. A number of studies have linked steroid resistance in asthma to defects in glucocorticoid receptor (GR) expression and activity, including reduced GR expression, reduced GR binding affinity to glucocorticoids and/or glucocorticoid response elements GREs, and elevated expression of pro-inflammatory transcription factors. Increased expression of IL-2, IL-4, and IL-13 in the airways of asthmatics can induce local steroid insensitivity by reducing GR binding affinity in T cells [17]. Immune cells from CR patients expressed more changes at the gene level as a result of an inadequate response to treatment. The data showed up-regulation of several genes related to the TNF signaling pathway, inflammatory response, cytokines, immune response, chemokine interleukin-8-like domain, and the NF-κB signaling pathway. Interestingly, we observed that TNF and inflammatory responses were among the top enriched pathways in patients who are resistant to corticosteroid treatment. Our data suggested that in the resistant patients, signaling via the corticosteroid pathway is defective; therefore, signaling through the TNF and inflammatory pathways remained active and were not suppressed in those patients. TNF was found to be involved in the development of allergic diseases, particularly asthma [18]. *TNF-α* is unlikely to be detected in healthy individuals; however, elevated levels in serum and tissue were found in inflammatory and infectious conditions [19,20]. Monocytes are an important source of TNF [21]. In addition, chemokines secreted by monocytes, e.g. CXCL2 [22], or chemoattractants for monocytes, CCL4 [23], were both elevated in CR, implying a plausible role of monocytes in the pathology of asthma.

The GSE19301 dataset compared gene expression changes in PBMCs during stable asthma, exacerbation, and 2 weeks after exacerbation (follow-up). At the onset of asthma exacerbation, only two genes were up-regulated compared with the stable state of asthma; 2 weeks later an additional three genes were up-regulated. Examining changes in the gene expression in the follow-up samples showed up-regulation of ten genes in PBMCs compared with stable asthma. Therefore, asthma exacerbation leads to changes in gene expression, whether immediately or 2 weeks later, compared with the stable state of asthma. Both of the common up-regulated genes, *IFI27* that is expressed by monocytes [24], and *IFI44L*, are interferon-response genes [25], and are thus inflammation-related genes [26,27]. However, the down-regulated gene encodes LTF, which exhibits an anti-inflammatory activity through its ability to inhibit eosinophil migration and regulates cellular growth and differentiation [28]. In addition, defensins are a family of antimicrobial and cytotoxic peptides involved in host defense that are expressed predominantly in neutrophils and epithelial cells [29], implying that asthma exacerbation induces an ongoing inflammatory response.

In the last study (GSE40240), the effect of allergen inhalation challenge on peripheral blood from asthmatic early responders and dual responders was assessed using gene expression analysis of blood collected from patients with asthma immediately before, and 2 hours after, cat allergen challenge. The findings of the present study implied a stronger effect of allergen exposure on dual responders compared with that in early responders because they exhibited more changes in gene expression immediately before, and 2 hours after, allergen exposure. In addition, more up-regulated genes were observed in the present study, which was compatible with the allergen challenge response strength. As time passed after the allergen challenge, fewer genes remain down-regulated, indicating that time affects the down-regulated genes more than the up-regulated genes.

In conclusion, our data unraveled gene signatures and signaling pathways in mononuclear cells correlating with asthma, severity, response to allergens, and response to corticosteroid treatment. Our data also highlight the multifactorial nature of asthma, and suggests that genes involved in asthma progression are different from those involved in the response to corticosteroids, with potential implications in the clinical management of different groups of asthmatic patients.

## Abbreviations

BAL: bronchoalveolar lavage
FEV1: forced expiratory volume 1
PBMC: peripheral blood mononuclear cell
TGF-β: transforming growth factor-β
TNF: tumor necrosis factor
